# Comparative whole-genome sequence analysis of *Mycobacterium tuberculosis* isolated from pulmonary tuberculosis and tuberculous lymphadenitis patients in Northwest Ethiopia

**DOI:** 10.3389/fmicb.2023.1211267

**Published:** 2023-06-30

**Authors:** Daniel Mekonnen, Abaineh Munshea, Endalkachew Nibret, Bethlehem Adnew, Silvia Herrera-Leon, Aranzazu Amor Aramendia, Agustín Benito, Estefanía Abascal, Camille Jacqueline, Abraham Aseffa, Laura Herrera-Leon

**Affiliations:** ^1^Department of Medical Laboratory Sciences, School of Health Science, College of Medicine and Health Sciences, Bahir Dar University, Bahir Dar, Ethiopia; ^2^Health Biotechnology Division, Institute of Biotechnology, Bahir Dar University, Bahir Dar, Ethiopia; ^3^Amhara Public Health Institute, Bahir Dar, Ethiopia; ^4^Department of Biology, Bahir Dar University, Bahir Dar, Ethiopia; ^5^Armauer Hansen Research Institute, Addis Ababa, Ethiopia; ^6^National Centre for Microbiology, Instituto de Salud Carlos III, Madrid, Spain; ^7^Fundación Mundo Sano, Madrid, Spain; ^8^National Center of Tropical Medicine, Institute of Health Carlos III, Centro de Investigación Biomédica en Red de Enfermedades Infecciosas, Madrid, Spain; ^9^European Public Health Microbiology Training Programme, European Centre for Disease Prevention and Control, Stockholm, Sweden; ^10^CIBER Epidemiologia y Salud Publica, Madrid, Spain

**Keywords:** *Mycobacterium tuberculosis*, tuberculous lymphadenitis, pulmonary tuberculosis, whole-genome sequencing, Ethiopia

## Abstract

**Background:**

Tuberculosis (TB), caused by the *Mycobacterium tuberculosis* complex (MTBC), is a chronic infectious disease with both pulmonary and extrapulmonary forms. This study set out to investigate and compare the genomic diversity and transmission dynamics of *Mycobacterium tuberculosis* (*Mtb*) isolates obtained from tuberculous lymphadenitis (TBLN) and pulmonary TB (PTB) cases in Northwest Ethiopia.

**Methods:**

A facility-based cross-sectional study was conducted using two groups of samples collected between February 2021 and June 2022 (Group 1) and between June 2020 and June 2022 (Group 2) in Northwest Ethiopia. Deoxyribonucleic acid (DNA) was extracted from 200 heat-inactivated *Mtb* isolates. Whole-genome sequencing (WGS) was performed from 161 isolates having ≥1 ng DNA/μl using Illumina NovaSeq 6000 technology.

**Results:**

From the total 161 isolates sequenced, 146 *Mtb* isolates were successfully genotyped into three lineages (L) and 18 sub-lineages. The Euro-American (EA, L4) lineage was the prevailing (*n* = 100; 68.5%) followed by Central Asian (CAS, L3, *n* = 43; 25.3%) and then L7 (*n* = 3; 2.05%). The L4.2.2.ETH sub-lineage accounted for 19.9%, while Haarlem estimated at 13.7%. The phylogenetic tree revealed distinct Mtb clusters between PTB and TBLN isolates even though there was no difference at lineages and sub-lineages levels. The clustering rate (CR) and recent transmission index (RTI) for PTB were 30 and 15%, respectively. Similarly, the CR and RTI for TBLN were 31.1 and 18 %, respectively.

**Conclusion and recommendations:**

PTB and TBLN isolates showed no *Mtb* lineages and sub-lineages difference. However, at the threshold of five allelic distances, *Mtb* isolates obtained from PTB and TBLN form distinct complexes in the phylogenetic tree, which indicates the presence of *Mtb* genomic variation among the two clinical forms. The high rate of clustering and RTI among TBLN implied that TBLN was likely the result of recent transmission and/or reactivation from short latency. Hence, the high incidence rate of TBLN in the Amhara region could be the result of *Mtb* genomic diversity and rapid clinical progression from primary infection and/or short latency. To validate this conclusion, a similar community-based study with a large sample size and better sampling technique is highly desirable. Additionally, analysis of genomic variants other than phylogenetic informative regions could give insightful information. Combined analysis of the host and the pathogen genome (GXG) together with environmental (GxGxE) factors could give comprehensive co-evolutionary information.

## 1. Background

Tuberculosis (TB) is a chronic infectious disease with complex epidemiological characteristics (Abel et al., 2014; Barberis et al., 2017; Adhikari, 2022). It is an ancient disease causing illness and death in humans since the primordial times (Palomino et al., 2007). Over 10.9 and 1.4 million TB cases and deaths were reported in 2021 alone (WHO, 2022). *Mycobacterium tuberculosis* complex (MTBC), the etiological agents of TB, includes *Mycobacterium tuberculosis (M. tuberculosis, Mtb)* (Sakula, 1982)*, M. africanum* (de Jong et al., 2010), *M. bovis, M. microti, M. pinnipedii, M. orygis, M. mungi, and M. suricattae* (Gagneux, 2018). The human-adapted members of MTBC (*Mtb* and *M. africanum*) are further classified into nine lineages with distinct geographic structures (Coscolla et al., 2021).

Ethiopia is a country in East Africa with high TB incidence and a complex *Mtb* population genetic structure (Mekonnen et al., 2019c). This complex population genetic structure is likely shaped by the consecutive entry of strains through long trade across the red sea, human migration over the millennia (Comas et al., 2013, 2015), and the long-established co-evolutionary trajectory (May and Anderson, 1983). The incidence of tuberculous lymphadenitis (TBLN), which is clinically characterized by the inflammation of the lymph node (cervical, axillary, or inguinal) with or without TB constitutional symptoms (cough, weight loss, fever, and night sweats), is exceptionally high in Ethiopia (Berg et al., 2015). However, causal factors leading to a high incidence rate of TBLN in Ethiopia remain speculative. Factors including ethnicity, Mtb strain type, HIV co-infection (Firdessa et al., 2013; Berg et al., 2015; Mekonnen et al., 2019b), over diagnosis (Iwnetu et al., 2009), and spill-over transmission of *bovine TB* (Ameni et al., 2011; Tadesse et al., 2017) were excluded. On the other hand, female sex, younger age (Mekonnen et al., 2019b), delayed diagnosis (Asres et al., 2019), and rural residency (Mekonnen et al., 2022) demonstrated a significant association.

The controversy about scientific evidence for “the incidence of TBLN with no lung involvement” is the subject of ongoing debate (Ganchua et al., 2020). For instance, Yew and Lee (1995) and Deveci et al. (2016) concluded that TBLN without pulmonary TB (PTB) is rare. To accept the theory that TBLN can exist as a distinct localized form of TB without pulmonary involvement, pathogenesis pathways other than lymphohematogenous spread, such as via tonsils and adenoids, must be proposed (Yew and Lee, 1995; Deveci et al., 2016). Herath and Lewis (2014) support the above claims and found a higher rate of concomitant PTB among extrapulmonary TB (EPTB) patients but with normal chest X-ray findings. In contrast to the above findings, a higher number of TBLN cases without concurrent PTB has been reported by multiple studies. Briefly, in Polesky et al.'s (2005) study, out of 106 TBLN cases, only 16 (15%) had evidence of pulmonary involvement. Of the total TBLN cases, concomitant PTB was reported among 20.8% of TBLN cases (Qian et al., 2018). In another study by Bomanji et al. (2020), only 100 (28%) out of 358 EPTB patients showed pulmonary involvement. Nevertheless, to disprove either of the two scientific narratives, a clinical, radiological, and pathological investigation should be coupled with bacteriological methods.

Whole-genome sequencing (WGS) technology demonstrated an incredible potential for investigating the population's genomic structure and transmission clusters (Gardy et al., 2011; Walker et al., 2013; and Meehan et al., 2018). Core genome multilocus sequence typing (cgMLST) and single-nucleotide polymorphism (SNP) with 5–12 SNP or allelic differences were suggested for identifying epidemiological links with temporal scales (Gardy et al., 2011; Walker et al., 2013; and Meehan et al., 2018). Recent transmission index (RTI) is the measure of the severity of the *Mtb* epidemic and indicates the rapid progression of the infection into clinical illness (Tanaka and Francis, 2005; Tessema et al., 2013).

A few number of studies examined whether the high incidence rate of TBLN in Ethiopia was associated with *Mtb* lineages and sub-lineages (Firdessa et al., 2013; Ejo et al., 2021). These findings indicated that lineages and sub-lineages were identical between PTB and TBLN forms. Furthermore, the rate of clustering was also similar among the two clinical forms (10% for PTB and 11% for TBLN) (Firdessa et al., 2013). Additionally, a comparative literature review in Africa showed no difference with regard to lineages and sub-lineages between the two forms of TB (Mekonnen et al., 2019a). However, as can be seen from the figures in our previous research studies (Mekonnen et al., 2019b, 2022), TBLN showed a geographic pattern similar to the MTBC geographic structuring. To explain further, TBLN is mainly reported in countries having a high prevalence of geographically restricted specialist MTBC (sub)-lineages (Mekonnen et al., 2019a). Hence, we speculated the presence of MTBC genomics influences on TB clinical phenotypes. Hence, this study was designed to investigate the genomic diversity and transmission dynamics of *Mycobacterium tuberculosis* (*Mtb*) isolates obtained from TBLN and PTB cases in Northwest Ethiopia using WGS data.

## 2. Materials and methods

### 2.1. Study design and setting

**Figure 1:**
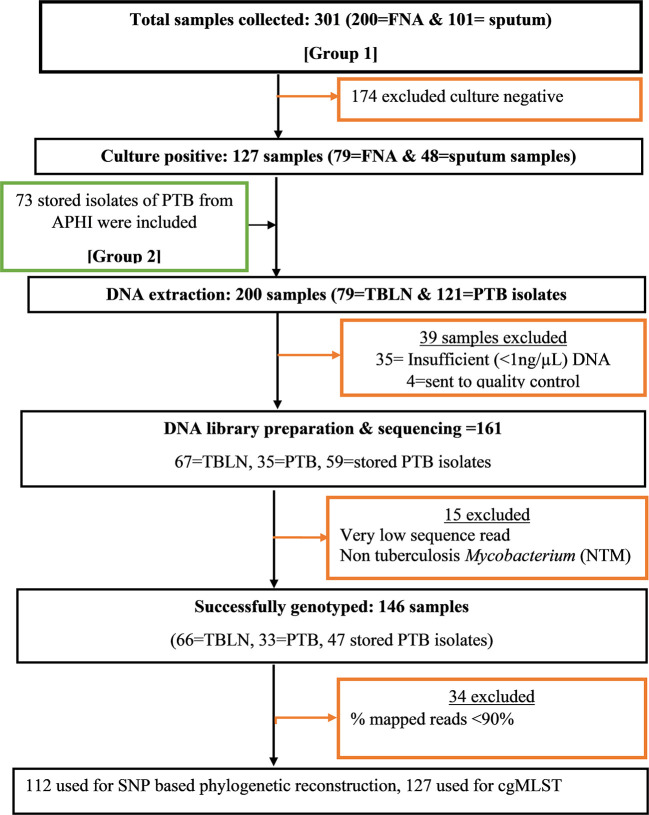
Flow diagram showing methodological workflow, Northwest Ethiopia, 2023. **Group 1:** Contain *Mtb* isolates obtained from new TB cases from both TBLN and PTB. **Group 2:** Contain Mtb isolates obtained from presumptive MDR-TB cases of PTB that were stored in Amhara Public Health institute Mycobacterium laboratory.

A facility-based cross-sectional survey was carried out with two sets of samples (new TB patients and presumptive MDR-TB cases). The first group of participants included new PTB and TBLN cases identified at outpatient departments in Bahir Dar health facilities (Felege-Hiwot Comprehensive Specialized Hospital/FHCSH, Bahir Dar Health Center, Han Health Center, and Shum-Abo Health Center) between February 2021 and June 2022. The second group included presumptive multidrug-resistant tuberculosis (MDR-TB) isolates archived at Amhara Public Health Institute's (APHI) *Mycobacterium* reference laboratory between June 2020 and June 2022 (Figure 1). Patients were selected from both urban and rural parts of Northwest Ethiopia.

### 2.2. *Mycobacterium tuberculosis* culture

At the APHI *Mycobacterium* reference laboratory, the stored isolates were re-grown using Löwenstein–Jensen (LJ) medium and *Mycobacterium* growth indicator tube (MGIT) media. When both LJ and MGIT gave confluent growth, the colony was taken from LJ, or otherwise, MGIT was used. When MGIT became positive, the culture was further incubated at 37°C for an additional 7 days to enrich the MTBC population and make the MTBC cells aggregate and clearly visible in the transparent media. One milliliter of MGIT media containing MTBC aggregates was transferred into 1.5-ml screw-cap vials. The MTBC grown on LJ media was harvested using a sterile plastic loop and transferred into 1.5-ml screw-cap vials containing 1 ml of distilled water. Then the colonies were inactivated by heating at 95°C for 30 min. The heat-inactivated MTBC was transported to the National Center of Microbiology, Institute of Carlos III (ISCIII), Madrid, Spain, for DNA extraction and WGS.

### 2.3. *Mycobacterium tuberculosis* DNA extraction

The DNA extraction was carried out using two methods: manual NZY Tissue gDNA isolation kit (*nzytech genes and enzymes, Lisboa, Portugal*) and robotic Maxwell RSC cultured cells DNA extraction kit (*Promega Biotech Ibérica S.L*.). DNA extraction using NZY tissue kit was performed according to the manufacturer's instructions with the following modifications: heat-inactivated MTBC isolate was centrifuged at 11,000 rpm for 2 min. After removing the supernatant, 180 μl of pre-aliquoted lysis buffer and 60 μl of reconstituted lysozyme (20 mg/ml) were added. The mixture was vortexed and incubated for 3 h at 37°C. Then 75 μl of sodium dodecylbenzenesulfonate (SDS) 10 × and 20 μl of proteinase K (20 mg/ml) were added, vortexed, and then incubated overnight at 56°C. The same pre-treatment was applied to the samples before DNA extraction using the robotic Maxwell RSC DNA kit-based extraction. The concentration of double-stranded DNA was measured using a fluorometer (Promega corporation), and the purity was measured using a NanoDrop spectrophotometer (Thermo Fisher Scientific) at a 260/280 wavelength absorbance ratio. A DNA quantity of ≥1 ng/μl was considered a cutoff value for library preparation. Figure 1 summarizes the key methods of the study.

### 2.4. Library preparation and sequencing

The input DNA library was prepared using Nextera DNA Flex Library Prep Workflow according to the manufacturer's protocol (Illumina, San Diego, CA, USA). Sequencing was carried out using Illumina NovaSeq 6000 technology (Modi et al., 2021).

### 2.5. Bioinformatics analysis

#### 2.5.1. Sequence read quality control and assembly

Quality control analysis was carried out using fastQC v.0.11.3 (http://www.bioinformatics.babraham.ac.uk/projects/fastqc/) and Trimmomatic v.0.36 software (Bolger et al., 2014) by the Bioinformatics Unit at the ISCIII. Assembly of the reads was performed using SPAdes v.3.8.0 (Bankevich et al., 2012), and the quality of the assembly was evaluated using QUAST software (Gurevich et al., 2013).

#### 2.5.2. Mapping and genotyping

Mapping was performed with MTBseq 1.0.3 (Kohl et al., 2018b) bioinformatic tool using Mtb RefSeq (GCF_003287165.1_ASM328716v1). Briefly, mapping was done using Burrows–Wheeler Aligner-Maximal Exact Match (BWA-MEM) algorithm. Sequence Alignment/Map tools (SAM tools) were used for sorting, indexing, removing putative PCR duplicates, and removing temporary files. Then, Genome Analysis Toolkit (GATK) was used for base call recalibration and realignment of reads around insertions or deletions. Later, variant calling/discovery and parsing were done for strains and group calling.

With default settings, variants need to be indicated by four reads mapped in each forward and reverse orientation, respectively, at 75% allele frequency, and by at least four calls with a phred quality score of at least 20. The last sample-specific module enables the phylogenetic classification of the input sample(s) according to Homolka et al. (2012) and Coll et al. (2014) SNP-based typing. In SNP-based barcoding, variant subsets are automatically generated and filtered for repetitive regions and resistance-associated genes (Kohl et al., 2018b).

#### 2.5.3. SNP and cgMLST-based phylogeny

Multiple sequence alignment (MSA) was carried out using multiple alignments using fast fourier transform (MAFFT) (Katoh et al., 2002) against H37Rv (Gen Bank accession number NC_000962.3) which can be assessed at https://www.ncbi.nlm.nih.gov/nuccore/NC_000962.3). Then, the SNP-based phylogenetic reconstruction was carried out using randomized axelerated maximum likelihood (RaxML) using the general time reversal (GTR) model and 1,000 bootstrap value (Stamatakis, 2006). Ancient *Mtb* strain was used as a root for the tree (Supplementary fasta file). Finally, the generated tree was edited using Figtree (https://github.com/rambaut/figtree/releases).

The cgMLST uses alleles as the unit of comparison, rather than nucleotide sequences. In allele-based comparisons among isolates, each allelic change is counted as a single genetic event, regardless of the number of nucleotide polymorphisms involved (Maiden et al., 2013). Assembled genomes were uploaded onto the Ridom SeqSphere software (version 8.5; Ridom GmbH, Münster, Germany). Each isolate sequence was aligned to the Ridom SeqSphere *Mtb* core genome scheme of 2,891 core genes which was obtained using the H37Rv reference genome (NC 000962.3) (Kohl et al., 2014). Two genes overlapping by more than four bases and genes with an internal stop codon in more than 80% of all query genomes were removed. Furthermore, repetitive genes such as PPE/PE-PGRS gene families were manually excluded from the scheme (Kohl et al., 2014). The result was generated as a minimum spanning tree and then edited using the Inkscape online tool v1.2 (https://inkscape.org/release/inkscape-1.2.2/).

Clustering rate (CR) and RTI were calculated from the minimum spanning tree complexes. A cluster was defined as two or more *Mtb* isolates differing with ≤ 5 alleles at core genomes (Gardy et al., 2011; Walker et al., 2013; and Kohl et al., 2018a). Isolates differing with ≤ 5 alleles at core genomic regions were considered transmission linked cases. The clustering rate and RTI were calculated using the following formula: CR% = (*n*/*N*)^*^100, RTI% = [(*n*-Nc)/*N*]^*^100, where *N* is the total isolates in the sample, *n* is the total number of isolates within the clusters, and Nc is the number of clusters (Tanaka and Francis, 2005; Tessema et al., 2013).

### 2.6. Statistical analysis

Descriptive statistics, such as mean, median, standard error (Std.error), and interquartile range (IQR), were used to summarize the evolutionary events (SNP, deletion, insertion, and substitution) of genotypes. Furthermore, an independent sample *t*-test was used to assess the correlation between TB forms (TBLN and PTB) and evolutionary events. Logistic regression analysis was carried out to identify factors associated with forms of TB (TBLN vs. PTB) and clustering (cluster vs. singleton). The analyses were carried out using R software version 4.2.2 (R Core Team, 2022) and the SPSS statistical software version 25 (*IBM Corp. Released 2017. IBM SPSS Statistics for Windows, Version 25.0. Armonk, NY: IBM Corp)*.

## 3. Results

Whole-genome sequencing was performed for 161 Mtb isolates using Illumina NovaSeq 6000 technology. The overall sequence read quality was good. The sequence read length varied from 35 to 151 nucleotides (mean 148). The mean and median sequencing coverage were 267 and 201 times, respectively. The mean (Std.error) and median (interquartile range/IQR) of SNP events were 1,163.62 (32.795) and 1,027.0 (498), respectively. The maximum deletion event among isolates was 610, and the minimum number of deletion was 114. The highest SNP, deletion, insertion, and substitution events were observed in L3 and L7 isolates than in L4 sub-lineages (Supplementary Table 1). An independent sample *t*-test was carried out to compare the mean evolutionary events between PTB and TBLN isolates. The analysis did not reveal any significant correlation (*p* > 0.05, Supplementary Table 2).

### 3.1. Genetic diversity of *Mycobacterium tuberculosis*

**Figure 2:**
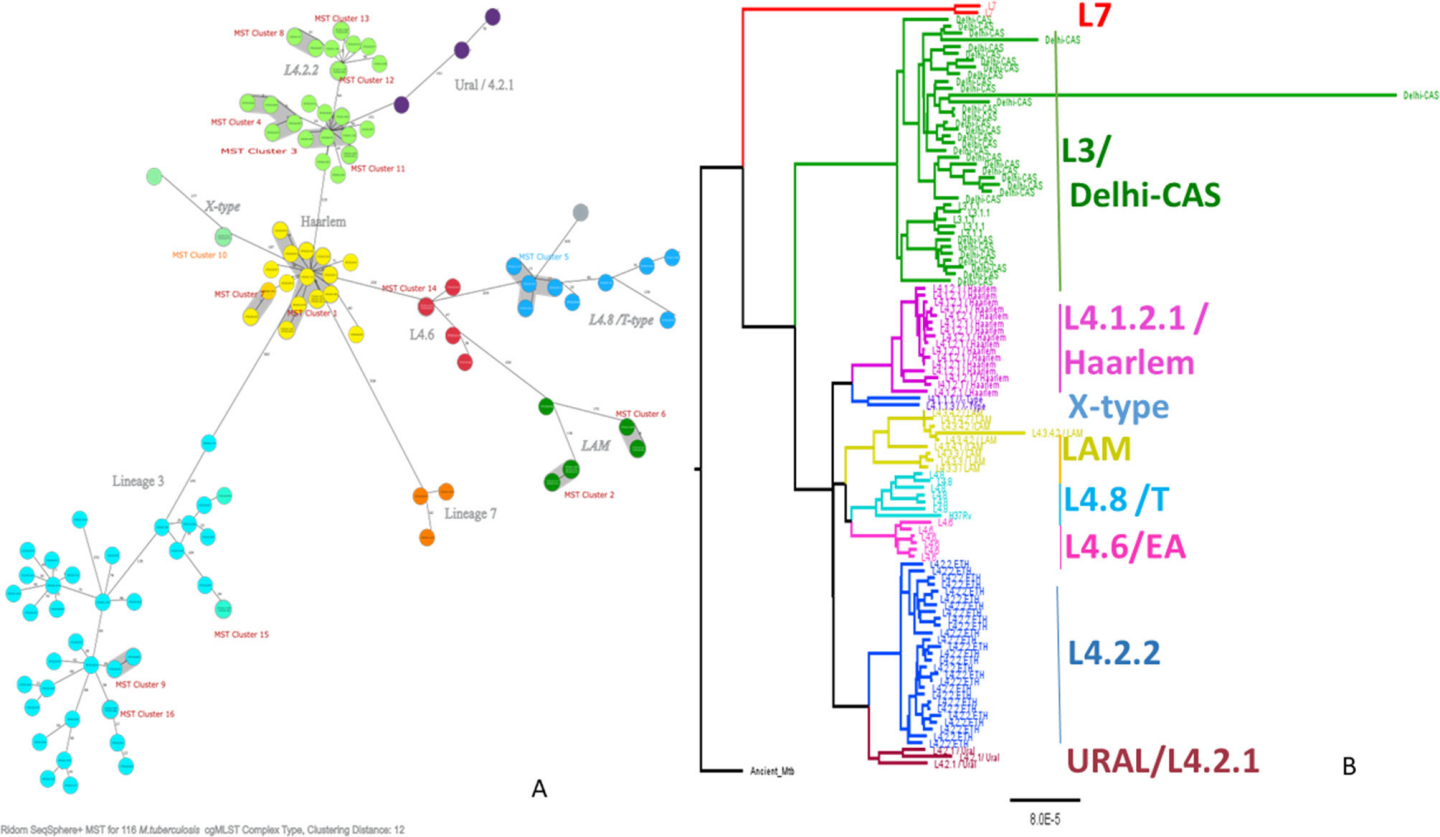
Minimum spanning tree showing cgMLST-based genetic distance **(A)**, maximum likelihood phylogenetic tree inferred from single nucleotide polymorphism **(B)** of Mtb isolated from PTB and TBLN cases in Northwest Ethiopia, 2023. CgMLST, core genome multilocus sequence typing; Mtb, Mycobacterium tuberculosis; SNP, single-nucleotide polymorphism.

According to Homolka et al. (2012) and Coll et al. (2014) SNP-based typing, 146 isolates were genotyped and classified within three lineages and 18 sub-lineages. Lineage 4 accounted for 100 (68.5%) of the 146 total isolates, while L3 accounted for 43 (25.3%). The remaining 3 (2.05%) isolates were shared by Lineage 7. Furthermore, L4.2.2/L4.2.2.ETH/sub-lineage was estimated to account for 19.9%, while the Haarlem/L4.1.2.1 accounted for 13.7%. The proportion and types of Mtb lineages and sub-lineages were comparable and/or the same between PTB and TBLN (Table 1). Briefly, from the total 37 Delhi-CAS sub-lineages, 21 (56.8%) and 16 (43.2%) Delhi-CAS sub-lineages were obtained from TBLN and PTB, respectively. Similarly, from 29 L4.2.2.ETH sub-lineages, 17 (58.6%) were from PTB and the rest 12 (41.4%) were from TBLN (Table 1; Figure 2).

**Table 1:** Mtb lineages (L) and sub-lineages and their distribution in PTB and TBLN cases, Northwest Ethiopia, 2023.

**Mtb genotypes**	**TBLN *N* (%)**	**PTB *N* (%)**	**Total (%)**
Delhi-CAS (L3)	21 (56.8)	16 (43.2)	37 (25.3)
Delhi-CAS (L3.1.1)	2 (33.3)	4 (66.7)	6 (4.1)
EA (L4.2)	0 (0.0)	1 (100.0)	1 (0.68)
EA (L4.6)	2 (28.6)	5 (71.4)	7 (4.8)
T (L4.8)	4 (44.4)	5 (55.6)	9 (6.2)
L7	2 (66.7)	1 (33.3)	3 (2.1)
X-type (L4.1.1.1)	0 (0.0)	4 (100.0)	4 (2.7)
X-type (L4.1.1.3)	0 (0.0)	2 (100.0)	2 (1.4)
Haarlem (L4.1.2.1)	9 (45.0)	11 (55.0)	20 (13.7)
Ural (L4.2.1)	3 (75.0)	1 (25.0)	4 (2.7)
EA.ETH (L4.2.2)	12 (41.4)	17 (58.6)	29 (19.9)
TUR (L4.2.2.1)	0 (0.0)	1 (100.0)	1 (0.68)
LAM (L4.3.3)	2 (66.7)	1 (33.3)	3 (2.1)
LAM (L4.3.4.1)	0 (0.0)	1 (100.0)	1 (0.7)
LAM (L4.3.4.2)	5 (100.0)	0 (0.0)	5 (3.4)
EA/T1 (L4.4.1)	3 (75.0)	1 (25.0)	4 (2.7)
EA/T1 (L4.4.1.2)	0 (0.0)	2 (100.0)	2 (1.4)
EA/T3 (4.6.2.1)	1 (12.5)	7 (87.5)	8 (5.5)
Total	66 (45.2)	80 (54.8)	146 (100.0)

As shown in Figure 2, L4.2.2, which was classified as L4.2.ETH (Comas et al., 2015), and spoligotyping international type (SIT149) (Firdessa et al., 2013; Yimer et al., 2015), is considered a specialist isolate in Ethiopia, formed two major groups in the tree. The Haarlem/L4.1.2.1 sub-lineages were positioned in the middle of the tree and contained the biggest cluster (cluster size: 13 isolates). Unlike EA sub-lineages, the population structure of L3 and L7 was deep-rooted, complex, and less clustered (Figure 2).

**Figure 3:**
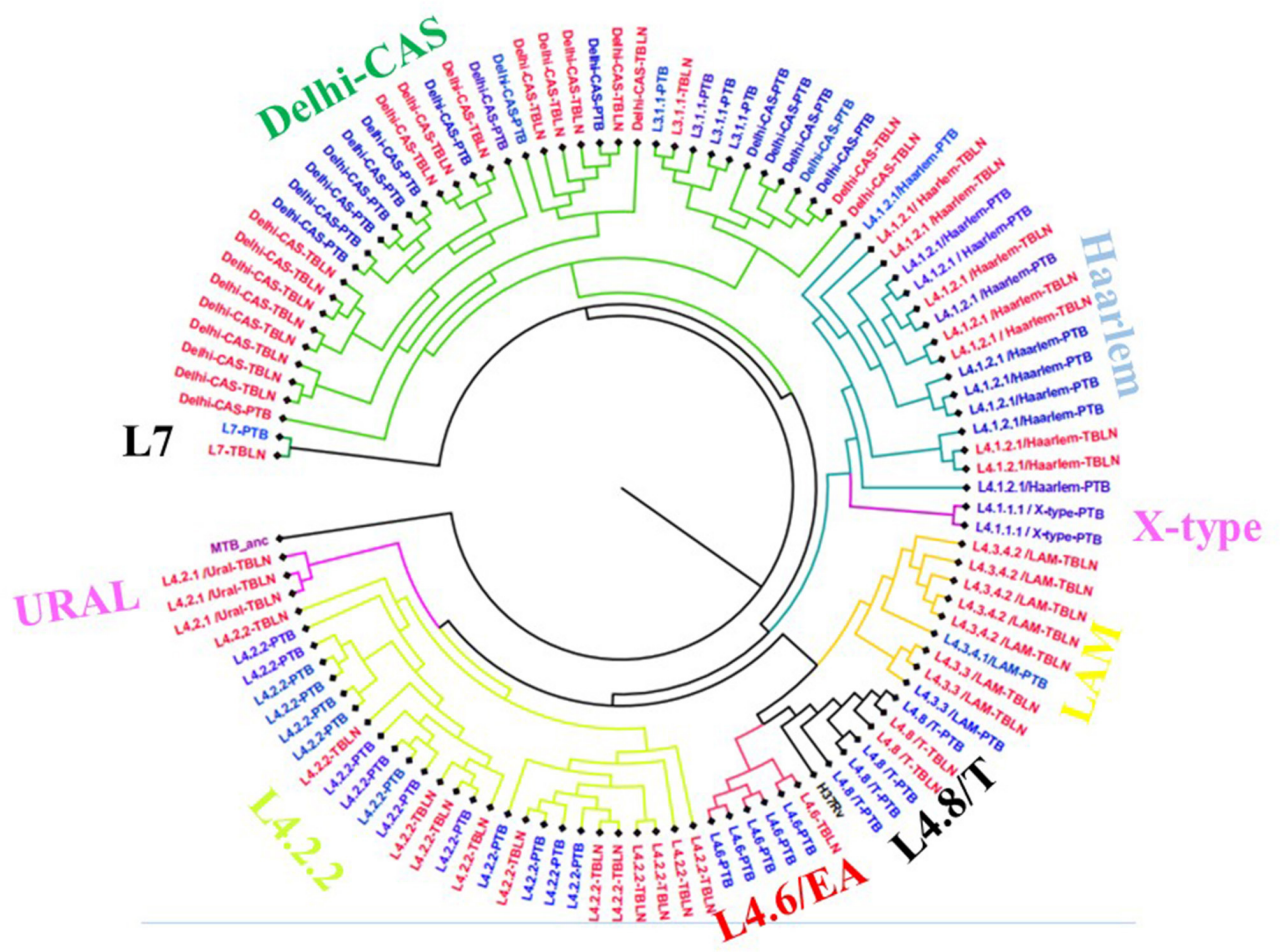
Comparative phylogeny of Mtb isolates obtained from PTB and TBLN, Northwest Ethiopia, 2023. Blue: PTB and Red: TBLN.

**Figure 4:**
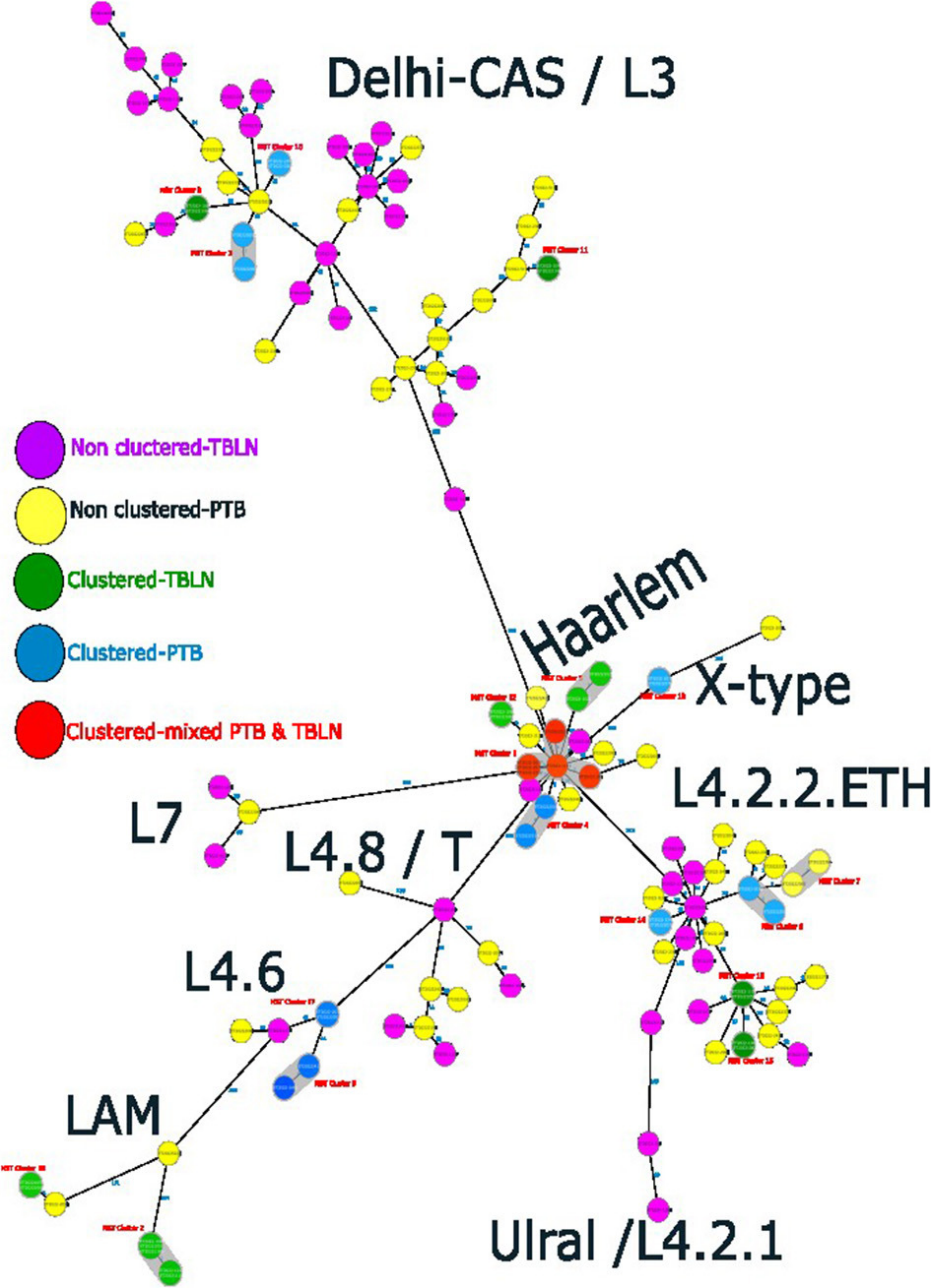
Ridom SeqSphere+ MST showing the distinct clusters of PTB and TBLN isolates at MST cluster distance threshold of 5, Northwest Ethiopia, 2023.

The comparative phylogenetic tree of Mtb isolates obtained from TBLN and PTB is depicted in Figures 3, 4. Surprisingly, unlike previous reports, these figures showed distinct Mtb genotypic clusters among PTB and TBLN isolates in Northwest Ethiopia. While several identical Mtb genotypes (isolates differing with < 5 alleles) cases were found from each of TBLN and PTB separately, a few identical Mtb isolates were identified from both PTB and TBLN (Figures 3, 4; Supplementary Table 3). A closer look at Figure 4; Supplementary Table 3 showed that PTB and TBLN shared one Haarlem genotype which is positioned centrally.

### 3.2. Transmission dynamics of *Mycobacterium tuberculosis*

**Figure 5:**
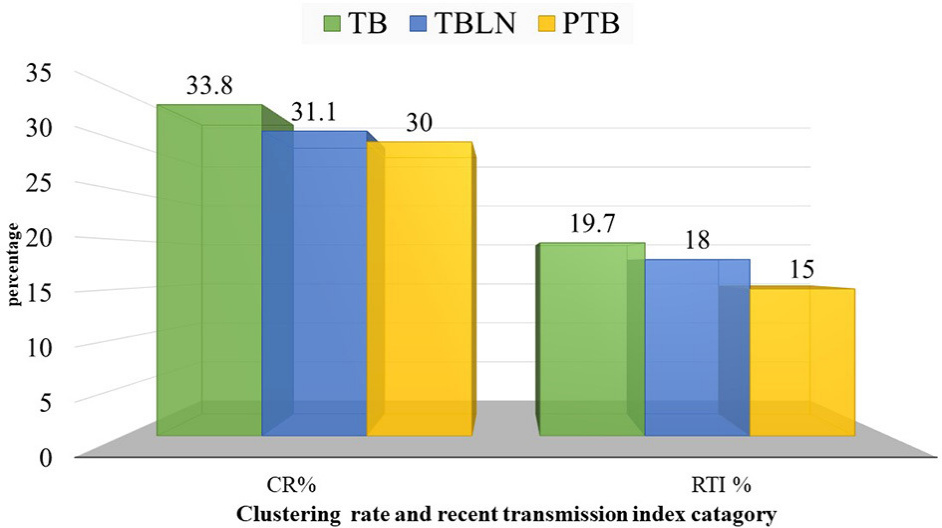
Clustering and recent transmission index disaggregated with forms of TB at a threshold of five allelic differences in Northwest Ethiopia, 2023.

At a threshold of five allelic distance, the cgMLST classified the 127 Mtb isolates into 84 cgMLST complex types, 84 unique and 18 clusters (Nc) containing 43 isolates (cluster size: 2–6 isolates). Thus, the overall CR and RTI were gauged at 33.8 and 19.7% (Figure 4; Supplementary Table 3), respectively. Furthermore, CR and RTI were calculated separately for PTB and TBLN. Hence the CR and RTI for PTB were 30 and 15% (number of isolates within the cluster = 18, Nc = 9, and *N* = 60), respectively. Similarly, the CR and RTI for TBLN were 31.1 and 18% (*n* = 19, Nc = 8, and *N* = 61), respectively. The second most surprising aspect of this study was the high RTI of TBLN (Figures 4, 5). Figure 5 depicts that the RTI of TBLN is unexpectedly high. The RTI is an indicator of epidemic severity, and it is the measure of the progression of clinical diseases from primary infection. The high RTI of TBLN implies that the TBLN is the result of recent transmission or reactivation from short latency. The higher proportion of TBLN among students (*p* > 0.05) was another evidence that the higher incidence of TBLN in Northwest Ethiopia is the result of recent transmission (Table 2).

**Table 2:** Logistic regression analysis TB forms with Mtb lineages and sub-lineages and socio-demographic factors, Northwest Ethiopia, 2023.

**Variables**		**TB forms**		***P*-value of COR**	**AOR (95%CI) *P*-value**
		**TBLN**	**PTB**		
Age groups (year)	≥65	3 (4.5)	0 (0)	1.00	–
	15–64	54 (81.8)	41 (100)	0.99	–
	1–14	9(13.6)	0 (0)	1	–
Gender	Female	33 (50.0)	19 (46.3)	0.713	0.698 (0.220–2.214) 0.541
	Male	33 (50.0)	22 (53.7)	1	1
Residency	Rural	38 (58.5)	12 (29.3)	**0.004**	**3.508 (1.085–11.40) 0.034**
	Urban	27 (41.5)	29 (70.7)	**1**	**1**
Marriage	Single	24 (38.7)	13 (40.6)	0.611	1.103 (0.199–6.120) 0.911
	Married	30 (48.4)	13 (40.6)	0.387	0.436 (0.097–1.963) 0.279
	Divorced and widowed	8 (12.9)	6 (18.8)	1	1
Current job	Pre-schooled children	2 (3.1)	0 (0)	0.99	–
	**Student**	**20 (31.3)**	**5 (15.6)**	**0.039**	**0.217 (0.038–1.232) 0.085**
	HW/farmer	24 (37.5)	8 (25.0)	0.07	0.907 (0.186–4.414) 0.903
	HRE	8 (12.5)	9 (28.1)	0.858	1.089 (0.215–5.521) 0.918
	Job-finder	10 (15.6)	10 (31.3)	1	1
Sub-lineages	EA	7 (10.6)	7 (16.7)	1.00	0.955 (0.137–6.642) 0.963
	L4.2.2.ETH	10 (15.2)	6 (14.3)	0.455	0.885 (0.128–6.124) 0.902
	Delhi- CAS	23 (34.8)	9 (21.4)	0.115	0.196 (0.036–1.075) 0.061
	Haarlem	9 (13.6)	8 (19.0)	0.858	0.939 (0.154–5.730) 0.946
	LAM	7 (10.6)	2 (4.8)	0.172	0.102 (0.006–1.619) 0.106
	Other	10 (15.2)	10 (23.8)	1	1

Bold values are either statistically significant or are higher within TBLN despite statistical insignificance.

Taken together, the Mtb isolates obtained from PTB and TBLN form distinct clusters which indicated the presence of strain genetic variation between the two clinical forms. Additionally, unlike the common belief that TBLN is mainly the result of reactivation of latent TB, this data demonstrated that TBLN is the result of recent transmission and/or rapid progression from short latency among immunocompetent adults.

The logistic regression analysis was computed using data from newly diagnosed PTB and TBLN cases (Group I: participants from Figure 1). The goodness of fit test, which is the measure of the ability of a model to generate high-quality predictions of the multivariable analysis, was assessed using the pseudo *R*^2^ and the Hosmer and Lemeshow test. It was found that the *p*-value of pseudo *R*^2^ and Hosmer and Lemeshow test were 0.54 and 0.797, respectively. Additionally, the percent correct classification of the model became 80.6%. These results indicated that the model was fairly good. Hence, L4.2.2.ETH with AOR: 7.42, 95%CI: 1.050–52.456 and a *p*-value of 0.045 and Haarlem with AOR: 27.37, 95%CI: 3.337–224.507 and a *p*-value of 0.002 demonstrated significant association with clustering. The two forms of TB (PTB vs. TBLN) did not show a significant association with clustering (*p* > 0.05). Moreover, other variables, such as gender, age, residency, and marital status, were not associated with Mtb clustering (Table 3).

**Table 3:** Logistic regression for clustering status with socio-demographic factors and Mtb sub-lineages, Northwest Ethiopia, 2023.

**Variables**		**Clustering status**		***P*-value of COR**	**AOR (95% CI) *P*-value**
		**Unique** ***N*** **(%)**	**Clustered** ***N*** **(%)**		
Age category (year)	1–14	5 (55.6)	4 (44.4)	0.99	–
	15–64	55 (57.9)	40 (42.1)	0.99	–
	≥65	3 (100)	0 (0)	1	1
Gender	Female	32 (61.5)	20 (38.5)	0.587	1.43 (0.413–4.985) 0.570
	Male	31 (56.4)	24 (43.6)	1	1
TB types	PTB	38 (57.6)	28 (42.4)	0.841	0.81 (0.233–2.847) 0.748
	TBLN	25 (59.5)	17 (40.5)	1	1
Residential status	Urban	33 (58.9)	23 (41.1)	0.911	0.51 (0.139–1.914) 0.322
	Rural	30 (60)	20 (40)	1	1
Marital status	Single	17 (45.9)	20 (54.1)	0.477	0.68 (0.113–4.047) 0.668
	Married	26 (60.5)	17 (39.5)	0.826	0.77 (0.147–3.994) 0.752
	Divorced and widowed	8 (57.1)	6 (42.9)	1	1
Current work	Pre-school	0 (0)	2(100)	0.99	–
	Student	12 (48)	13 (52)	0.894	1.35 (0.223–8.208) 0.743
	HW/farmer	21 (65.5)	11 (34.4)	0.266	0.22 (0.034–1.410) 0.110
	HRE	10 (58.8)	7 (41.2)	0.592	0.83 (0.158–4.362) 0.826
	Job-finder	10 (50)	10 (50)	1	1
Mtb sub-lineage	EA	11 (78.6)	3 (21.4)	0.809	1.29 (0.160–10.434) 0.811
	L4.2.2.ETH	7 (43.8)	9 (56.3)	0.061	**7.42 (1.050–52.456) 0.045**
	CAS	26 (81.3)	6 (18.8)	0.592	1.54 (0.270–8.736) 0.628
	Haarlem (4.1.2.1)	3 (17.6)	14 (82.4)	0.001	**27.37 (3.337–224.507) 0.002**
	LAM	1 (11.1)	8 (88.9)	0.007	–
	Other	15 (75)	5 (25)	1	1
Total	**63 (58.3)**	**45 (41.7)**		

Mtb, *Mycobacterium tuberculosis*; COR, crude odds ratio; AOR, adjusted odds ratio; PTB, pulmonary tuberculosis; TBLN, Tuberculosis lymphadenitis; HRE, high-risk employee; HW, housewife; EA.ETH, Euro-American lineage restricted to Ethiopia; LAM, Latin America- Mediterranean; T, Tuscany; Delhi-CAS, Delhi-Central Asia; Other, T, X, Ural, Uganda. Bolded variables are statistically significant variables.

## 4. Discussion

A few studies used the WGS technique to determine the genomic diversity and transmission dynamics of Mtb in Ethiopia (Comas et al., 2015; Yimer et al., 2016; and Welekidan et al., 2021). However, the aims of these studies were different from each other and also different from the present study. This study assessed and compared the genomic diversity and transmission dynamic of Mtb isolates obtained from PTB and TBLN cases in Northwest Ethiopia.

The WGS data unveiled a high genetic diversity of the Mtb population, L4 being the predominant (100/146, 68.5%), followed by L3 (29.45%). While the LAM and Haarlem isolates showed a low rate of SNP, insertion, substitution, and deletion, the L3 and L7 isolates demonstrated a high rate of mutations (Supplementary Table 1). The difference might be due to the nature (sympatric vs. allopatric) and timing of the pathogens–host association. When the specific host and Mtb coevolve together for a long, the host presents constant metabolic supplies. Thus, the metabolic genes of the Mtb become rendered useless and deleted (Ochman and Moran, 2001; Moran, 2002) as a way to cellular economization (Martínez-Cano et al., 2015).

**Figure 6:**
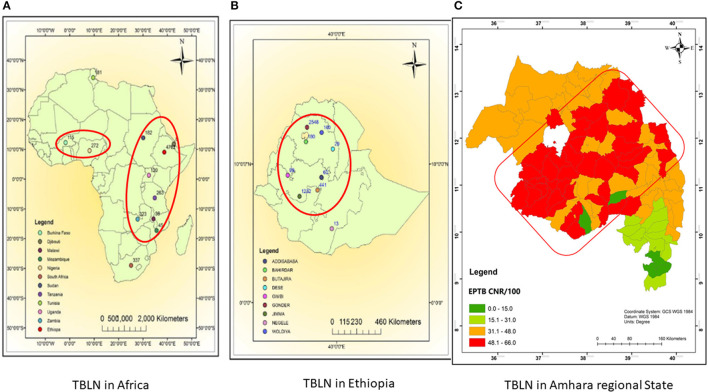
Map showing TBLN geographic distribution in Africa **(A)**, Ethiopia **(B)**, and Amhara regional State **(C)**, 2023. **(A, B)** were taken from Mekonnen et al. (2019b) and **(C)** was taken from Mekonnen et al. (2022) with acknowledgment.

The comparative Mtb genetic diversity showed similar lineages and sub-lineages, but it showed distinct genomic clusters among the two clinical forms (PTB vs. TBLN). The clusters became mixed of TBLN and PTB isolates at a higher threshold of allelic difference (>5 allelic difference). Faksri et al.'s (2018) study found genetic variants of Mtb that are commonly found in TB meningitis patients compared to PTB cases. To further support our finding, we re-evaluated the TBLN geographic distributions mapped in the previous studies at Africa (Mekonnen et al., 2019b), Ethiopia (Mekonnen et al., 2019b), and Amhara Regional State level (Mekonnen et al., 2022). Figures 6A, B shows the geographic pattern of TBLN similar to Mtb geographical structuring. Furthermore, we assessed the Mtb lineages and sub-lineages distribution among countries that had TBLN reports (Mekonnen et al., 2019a). As such, TBLN was mainly reported from West and East African countries, having a complex MTBC population genetic structure and high prevalence of Mtb Genotypes that have narrow host ranges (specialist genotypes). For instance, in West Africa, *L5/6/Maf* and L4.6.2/LAM-CAM are dominant specialist lineages and sub-lineages, respectively. Similarly in East Africa, L4.6/SIT37, L4.2.2/SIT149, L4.10/SIT53, L4.6.1/T2_Uganda, and several CAS sub-lineages are prevalent (Mekonnen et al., 2019a). Hence, the high incidence rate of TBLN in Ethiopia and other Eastern and Western African countries is likely the result of Mtb genomic variation. Negrete-Paz et al. (2021) also demonstrated intra-(sub)-lineage differences among TB clinical phenotypes. They briefly stated that strains of a sub-lineage vary in terms of immune response and virulence. Hence, these variations perhaps affect disease severity and clinical presentation (Negrete-Paz et al., 2021). Tuberculous lymphadenitis is the less severe form of TB (Hasan et al., 2009). It is known that long-established host–parasite coevolution leads to benign disease with insidious onset (May and Anderson, 1983). Further computational co-evolution model intuition could prove this scenario if it works for TBLN.

Unlike other zones of Amhara Regional State shown in Figure 6C, the low incidence rate of TBLN in the North Shewa Zone (Figure 6C light green color) could be due to environmental and host genetic differences besides Mtb genomics. Unlike other zones of Amhara, North Shewa is high land with cold weather.

Perhaps the other most compelling finding of this study is the higher CR and RTI in TBLN cases which are even slightly higher compared with PTB cases (*p* > 0.05). This is direct evidence showing that the high incidence rate of TBLN in the Amhara region is not just a result of the reactivation of latent TB. Rather, the finding indicated the rapid progression of clinical illness from primary infection or short latency. The high incidence rate of TBLN in younger age people, such as students (Table 2), was another evidence of the short latency nature of TBLN in Amhara Regional State. This rapid clinical progression of TBLN from primary infection was not due to immunodeficiency. This is because the immunohematological values of TBLN are relatively higher among TBLN compared with PTB (Mekonnen et al., 2023). Rather, it might be due to immunological tolerance resulting from sympatric-host pathogen association (Seal et al., 2021).

Due to the poor health system and socio-demographic factors, unrecognized extensive TB transmission might have taken place in Ethiopia for a long period. Such hidden extensive micro epidemics could probably push the co-evolution toward immune tolerance and then TBLN clinical phenotype. Host controls Mtb infections by activating innate and adaptive cellular immunities. Immune response against Mtb causes immunopathology in both the host and the pathogen. Hence, the host's ability to invest in self-toxic immune responses might have a limit. Beyond this threshold, the host switches from active resistance to tolerance strategy. Furthermore, tolerance to invading pathogens also has a threshold. Hence, the host's immune response strategy against Mtb is perhaps fine-tuned by the fitness effects of both the host and Mtb. This ability of the host for maintaining a balanced immunity and homeostasis during infection results in less severe forms of TB, such as TBLN, smear-negative PTB, lower cavitation, and negative chest X-ray finding (Seal et al., 2021). Taken together, the insights gained from this study may be of assistance to re-evaluate the current prevailing dogma which says- “no strain difference between PTB and TBLN isolates.”

L4.2.2.ETH forms two major branches containing a mix of clustered and unique strains (Figure 2). The L4.2.2.ETH topology in the cgMLST and SNP phylogeny is similar to Comas et al.'s (2015) phylogenetic tree. The phylogenetic position of L4.2.2.ETH is close to the Ural family, indicating their introduction into Ethiopia from Central Asian states (Mokrousov, 2012). Compared with L4.6, the arrival of L4.2 into Ethiopia is likely a recent phenomenon and possibly linked with the major north–south human migration during the reign of the Queen of Sheba (Comas et al., 2015). The LAM (L4.3.4.1 & L4.3.4.2) sub-lineages showed low deletion and substitution rates in this study. LAM is considered a generalist sub-lineage that is found in over 47 countries and highly prevalent in Europe with high genetic diversity. Its global success was likely driven by European migration and colonization (Stucki et al., 2016). The other highly clustered sub-lineage was Haarlem/L4.1.2.1 which is found in over 49 countries (Stucki et al., 2016). This sub-lineage is estimated to be introduced into Ethiopia at the time of early Portuguese contact with Ethiopia in the 16th century or later (Comas et al., 2015).

Lineage 3 is the second leading genotype found in this study, and this report is in line with previous studies (Tessema et al., 2013; Biadglegne et al., 2015; Mekonnen et al., 2019c; and Welekidan et al., 2021). There is a high genomic substitution, deletion, or SNP in L3 similar to L7. Our phylogenetic tree showed that the genetic diversity of L3 is very complex and likely shaped by several independent evolutionary events or multiple entries into Ethiopia (Figure 3). The L3 contains both specialist (isolates restricted in some countries including Ethiopia) and generalist genotypes (Comas et al., 2015; Tulu and Ameni, 2018). With a 90.2% probability, South Asia was predicted as the origin of all L3 strains with multiple (at least four) independent introductions into East and North Africa (Shuaib et al., 2022). L7 was restricted to Ethiopia and branched off around the time of initial human migrations out of Africa (Comas et al., 2015). Collectively, except L7, the other (sub) lineages followed the human migration; out of Africa (northern route via Egypt) (Pagani et al., 2015) and back migration into Africa in the different demographic histories (Pankhurst, 1964; Molinaro et al., 2019). Taken together, the population genomic structure of Mtb in Ethiopia is likely driven by long and stable host-Mtb reciprocal evolutionary changes (May and Anderson, 1983; Freschi et al., 2021) and consecutive entry of new variants via trade, missionaries, and human migration (Curtis, 2008; Comas et al., 2015). In addition to that, the poverty and high population density might have contributed to local adaptation and expansion of newly imported Mtb.

## 5. Strength and limitations

This WGS study gave high-resolution information about the role of Mtb genotypes in the clinical presentation of TB, genomic diversity, and transmission dynamics. However, it has some limitations. This study was carried out at the institutional level and included patients coming from different zones. Thus, clustered isolates did not mean they are epidemiologically linked, rather, it might be due to the widespread availability of the genotypes in the regional state. Similarly, the unique isolates might not be the result of reactivation, rather it might have been linked to cases in the community where the patient came. Hence, there might be a sampling bias. Furthermore, the sample size was small which precluded us from the in-depth assessment of the socio-demographic-related variables.

## 6. Conclusion and recommendations

TBLN and PTB showed no difference at lineage and sub-lineage levels. However, the isolates from the two clinical forms formed a distinct cluster, indicating the presence of pathogen genomic factors. The higher rate of clustering and RTI among TBLN confirmed the rapid progression of TBLN from primary infection and/or short latency. Hence, the high incidence rate of TBLN in Amhara Regional State is likely shaped by Mtb genetic diversity and rapid progression from primary infection. Comparative community-based studies containing data from the environment and host genome could give a detailed view. Additionally, the characterization of lineage-independent genetic components might give further insight.

## Data availability statement

The original contributions presented in the study are publicly available. This data can be found here: NCBI, PRJNA975069.

## Ethics statement

The study was approved by research and Ethical Review Committee of Science College of Bahir Dar University, with reference number PGRCSV/111/2012. Informed written consent was obtained from each participant before data collection. This study was conducted in accordance with the Declaration of Helsinki. All the information obtained from the study subjects were coded to maintain confidentially. The patients/participants provided their written informed consent to participate in this study.

## Author contributions

DM: substantial contributions to the conception of the work, data collection, laboratory works, analyses, interpretation of data for the work, and drafting of the work. AM and EN: substantially contributed to the conception, methodology, validation, monitoring of the work, secured partial fund for the work, and revising it critically for important intellectual content. BA: substantially contributed to the bioinformatics analysis and revised it critically for important intellectual content. AAA and AB: substantially contributed to supervision, monitoring of the laboratory work, and revising it critically for important intellectual content. EA and SH-L: participated in the DNA extraction, data analysis and interpretation, and revising the draft critically for important intellectual content. CJ: prepared the sequencing library, substantially contributed to the data analyses, interpretation of data for the work, and revised the draft critically for important intellectual content. AA: substantially contributed to the conception, methodology, and revising the draft critically for important intellectual content. LH-L: monitoring and coordinating the DNA extraction and WGS, substantially contributed to the bioinformatics, and revising the draft critically for important intellectual content. All authors critically revised and approved the version to be published.

## 7. Glossary

AOR: Adjusted odds ratio
APHI: Amhara Public Health Institute
BWA-MEM: Burrows–Wheeler Aligner-Maximal Exact Match
CAS: Central Asia
cgMLST: core genome multilocus sequence typing
COR: crude odds ratio
DRTB: drug-resistant TB
EA.ETH: Euro-American lineage restricted to Ethiopia
EA: Euro-American
EAI: East African Indian
EPM: enhanced PCR mix
GATK: Genome Analysis Toolkit
HRE: high-risk employee
HW: housewife
L: lineages
LAM: Latin America-Mediterranean
LJ: Löwenstein–Jensen medium
Mtb: *Mycobacterium tuberculosis*
MDR-TB: multidrug-resistant TB
MGIT: Mycobacterium growth indicator tube
MRCA: most recent common ancestor
MSA: multiple sequence alignment
MTBC: *Mycobacterium tuberculosis complex;*
NR: Neolithic revolution
PTB: pulmonary tuberculosis
RaxML: randomized axelerated maximum likelihood
SDS: sodium dodecylbenzenesulfonate
SEA: South East Asia
SNP: single-nucleotide polymorphism
T: Tuscany
TB: tuberculosis
TBL: terminal branch lengths
TBLN: *Tuberculosis lymphadenitis*
WGS: whole-genome sequencing
WHO: World Health Organization
XDR-TB: extensively drug-resistant TB.
