# Supplementary material for: Comparative whole-genome sequence analysis of Mycobacterium tuberculosis isolated from pulmonary tuberculosis and tuberculous lymphadenitis patients in Northwest Ethiopia
Source: Front Microbiol. 2023 Jun 30;14:1211267. doi: 10.3389/fmicb.2023.1211267 (PMC10348828; doi:10.3389/fmicb.2023.1211267)
Supplement: Supplementary file 3 [file Table_2.docx]

Supplementary Table 2. Correlation of *M. tuberculosis* evolutionary events with tuberculosis clinical presentation Northwest Ethiopia, 2023

| Ranks | | | | | Test Statistics | | | |
| --- | --- | --- | --- | --- | --- | --- | --- | --- |
| Evolutionary events | TB_Forms | N | Mean Rank | Sum of Ranks | Mann-Whitney U | Wilcoxon W | Z | Asymp. Sig. (2-tailed) |
| SNPs | TBLN | 51 | 54.95 | 2802.50 | 1073.5 | 2348.5 | -1.369 | .171 |
|  | PTB | 50 | 46.97 | 2348.50 |  |  |  |  |
| Deletions | TBLN | 51 | 54.47 | 2778.00 | 1098.0 | 2373.0 | -1.202 | .229 |
|  | PTB | 50 | 47.46 | 2373.00 |  |  |  |  |
| Insertions | TBLN | 51 | 51.66 | 2634.50 | 1241.5 | 2516.5 | -.228 | .820 |
|  | PTB | 50 | 50.33 | 2516.50 |  |  |  |  |
| Substitutions (Including Stop Codons) | TBLN | 51 | 54.43 | 2776.00 | 1100.0 | 2375.0 | -1.189 | .235 |
|  | PTB | 50 | 47.50 | 2375.00 |  |  |  |  |

SNP: Single nucleotide polymorphism, TB: Tuberculosis, TBLN: tuberculous lymphadenitis, PTB: Pulmonary tuberculosis
