# Supplementary material for: Comparative whole-genome sequence analysis of Mycobacterium tuberculosis isolated from pulmonary tuberculosis and tuberculous lymphadenitis patients in Northwest Ethiopia
Source: Front Microbiol. 2023 Jun 30;14:1211267. doi: 10.3389/fmicb.2023.1211267 (PMC10348828; doi:10.3389/fmicb.2023.1211267)
Supplement: Supplementary file 4 [file Table_3.docx]

Supplementary material 3: The list of distinct clusters disaggregated by TB forms, Northwest Ethiopia, 2023

| Cluster | Sub-Lineage | Sample ID | Cluster of | | |
| --- | --- | --- | --- | --- | --- |
|  |  |  | PTB | TBLN | Both PTB & TBLN |
| 1 | L4.1.2.1/Haarlem | -2077,-2079,-2312,2011, 1111, 1188 |  |  | √ |
| 2 | L4.3.4.2 /LAM | -1017, 1045,1051,1110, 1160 |  | √ |  |
| 3 | L3/Delhi-CAS | 2001, 2082 | √ |  |  |
| 4 | L4.1.2.1 /Haarlem | 2014, 2019 | √ |  |  |
| 5 | L4.1.2.1/Haarlem | 1021,1025 |  | √ |  |
| 6 | L4.2.2.ETH | 2359,5182 | √ |  |  |
| 7 | L4.2.2.ETH | 2363,2365 | √ |  |  |
| 8 | L4.6 | 2410,2441 | √ |  |  |
| 9 | L3/Delhi-CAS | 1066, 1067 |  | √ |  |
| 10 | L3/Delhi-CAS | 2353,2362 | √ |  |  |
| 11 | L3.1.1 /Delhi-CAS | 1046,1146 |  | √ |  |
| 12 | L4.1.2.1 /Haarlem | 1061,1063 |  | √ |  |
| 13 | L4.1.1.1 /X-type | 2013, 2076 | √ |  |  |
| 14 | L4.2.2.ETH | 2022,2348 | √ |  |  |
| 15 | 4.2.2.ETH | 1058,1159 |  | √ |  |
| 16 | 4.2.2.ETH | 1082,1083 |  | √ |  |
| 17 | L4.6 | 2003,2031 | √ |  |  |
| 18 | L4.3.3/LAM | 1055,1060 |  | √ |  |

**PTB:** Pulmonary tuberculosis, **TBLN:** Tuberculous Lymphadenitis

In cluster 1, the red highlighted IDs are TBLN and the rest are for PTB isolates. The table shows the distinct clustering status of Mtb lineages and sub-lineages with regard to TB forms
